# IMPACT OF HOME AMBIENT TEMPERATURE ON SELF-REPORTED MOOD AND ATTENTION IN COMMUNITY-DWELLING OLDER ADULTS

**DOI:** 10.1093/geroni/igac059.3086

**Published:** 2022-12-20

**Authors:** Amir Baniassadi, Wanting Yu, Ryan Day, Angel Wong, Thomas Travison, Lewis Lipsitz, Brad Manor

**Affiliations:** Harvard Medical School, Boston, Massachusetts, United States; Hebrew SeniorLife, Roslindale, Massachusetts, United States; Hinda and Arthur Marcus Institute for Aging Research, Boston, Massachusetts, United States; Hinda and Arthur Marcus Institute for Aging Research, Boston, Massachusetts, United States; Harvard Medical School, Boston, Massachusetts, United States; Hebrew SeniorLife, Brookline, Massachusetts, United States; Hebrew SeniorLife/Harvard Medical School, Roslindale, Massachusetts, United States

## Abstract

**Background:**

Many older adults experience variations in daily mood and/or attention. Lab-based studies show that, among other variables, ambient temperature can influence both. The objective of this study was to determine if and how habitual home temperature influences self-reported mood and attention in this population.

**Methods:**

Ambient temperature and humidity data were collected from the homes of 41 community-dwelling older adults (age=78±7, 35 females) living in Boston from June 1st to Aug 15th. Participants received two time-stamped smartphone-based questionnaires each day to report their mood and attention.

**Results:**

On average, participants completed 86(±29) questionnaires. Those with most variations in subjective outcomes (top quartile of % of time reporting “feeling down/depressed” or “difficult keeping attention”), compared to the rest of the sample, tended to reside in homes with both higher mean ambient temperature (p=0.01) and greater deviation in temperature over time (p=0.10). Logistic regression analysis combining data from all participants revealed that ambient temperature at the time of response did not predict either self-reported outcome. However, within-subject analyses indicated that of the 17 participants who reported at least some variation in attention or mood, the likelihood of experiencing poor mood and/or attention was correlated with time-synced ambient temperature in six individuals. Gender, age, or housing type (affordable vs. private) did not predict the presence of such associations.

**Conclusion:**

Variations in self-reported mood and attention are at least partially explained by the home thermal environment in a non-trivial fraction of older adults.

